# Supplementary material for: A measurement strategy to address disparities across household energy burdens
Source: Nat Commun. 2022 Jan 12;13:288. doi: 10.1038/s41467-021-27673-y (PMC8755748; doi:10.1038/s41467-021-27673-y)
Supplement: Supplementary file 2 — Reporting Summary [file 41467_2021_27673_MOESM2_ESM.pdf]

nature portfolio

Corresponding author(s): NCOMMS-21-25017-T

Last updated by author(s): Jul 10, 2021

Reporting Summary

Nature Portfolio wishes to improve the reproducibility of the work that we publish. This form provides structure for consistency and transparency in reporting. For further information on Nature Portfolio policies, see our Editorial Policies and the Editorial Policy Checklist.

Statistics

For all statistical analyses, confirm that the following items are present in the figure legend, table legend, main text, or Methods section.

n/a	Confirmed	
☐	☒	The exact sample size (*n*) for each experimental group/condition, given as a discrete number and unit of measurement
☐	☒	A statement on whether measurements were taken from distinct samples or whether the same sample was measured repeatedly
☒	☐	The statistical test(s) used AND whether they are one- or two-sided<br>*Only common tests should be described solely by name; describe more complex techniques in the Methods section.*
☒	☐	A description of all covariates tested
☐	☒	A description of any assumptions or corrections, such as tests of normality and adjustment for multiple comparisons
☒	☐	A full description of the statistical parameters including central tendency (e.g. means) or other basic estimates (e.g. regression coefficient) AND variation (e.g. standard deviation) or associated estimates of uncertainty (e.g. confidence intervals)
☒	☐	For null hypothesis testing, the test statistic (e.g. *F*, *t*, *r*) with confidence intervals, effect sizes, degrees of freedom and *P* value noted<br>*Give P values as exact values whenever suitable.*
☒	☐	For Bayesian analysis, information on the choice of priors and Markov chain Monte Carlo settings
☒	☐	For hierarchical and complex designs, identification of the appropriate level for tests and full reporting of outcomes
☒	☐	Estimates of effect sizes (e.g. Cohen's *d*, Pearson's *r*), indicating how they were calculated

Our web collection on statistics for biologists contains articles on many of the points above.

Software and code

Policy information about availability of computer code

Data collection	The full (anonymized) source code for data collection and analysis has been submitted for peer review. The full source code is available at https://www.github.com/ericscheier/net_energy_equity.
Data analysis	The full (anonymized) source code for data collection and analysis has been submitted for peer review. The full source code is available at https://www.github.com/ericscheier/net_energy_equity.

For manuscripts utilizing custom algorithms or software that are central to the research but not yet described in published literature, software must be made available to editors and reviewers. We strongly encourage code deposition in a community repository (e.g. GitHub). See the Nature Portfolio guidelines for submitting code & software for further information.

Data

Policy information about availability of data

All manuscripts must include a data availability statement. This statement should provide the following information, where applicable:

- Accession codes, unique identifiers, or web links for publicly available datasets
- A description of any restrictions on data availability
- For clinical datasets or third party data, please ensure that the statement adheres to our policy

All data necessary for the composition of the source datasets used in this analysis are freely available from United States government sources as open data. All functions to automatically retrieve and assemble these data, and compiled versions of these data are made available to the user as part of the software referred to in the Code Availability Section.

Field-specific reporting

Please select the one below that is the best fit for your research. If you are not sure, read the appropriate sections before making your selection.

☐ Life sciences ☒ Behavioural & social sciences ☐ Ecological, evolutionary & environmental sciences

For a reference copy of the document with all sections, see nature.com/documents/nr-reporting-summary-flat.pdf

Behavioural & social sciences study design

All studies must disclose on these points even when the disclosure is negative.

Study description	The study examines summary statistics of quantitative estimates of household energy expenditures and incomes in the United States.
Research sample	The Low-Income Energy Affordability Dataset (LEAD) was assembled by the National Renewable Energy Laboratory (NREL) on behalf of the United States Department of Energy's (DOE) Clean Energy for Low-Income Communities Accelerator (CELICA) Toolkit. It estimates the household energy expenditures and incomes for all households in the United States.
Sampling strategy	Spatial allocation of different housing unit types relies on the use of an iterative proportional fitting (IPF) algorithm. IPF is used sequentially to build increasingly complex cross tabulations. Census tract-level published tables from the U.S. Census housing data from the 2016 5-year American Community Survey are used as the marginal totals, and cross-tabulations of the ACS5 Public Use Microdata Samples for the corresponding Public Use Microdata Areas are used as the seeds in the IPF algorithm.
Data collection	Public Use Microdata samples from the 2016 5-year American Community Survey are scaled to match aggregate annual values from utility sales and revenues reported in Energy Information Administration forms 861 and 176.
Timing	Start: 2012<br>Stop: 2016
Data exclusions	Approximately 23,184,292 of 136,384,292 households were excluded if they (a) were mobile homes such as trailers, boats, and recreational vehicles or (b) did not have any estimated energy expenditures (due to the inability to divide by 0 during the analysis). These criteria were pre-established.
Non-participation	N/A
Randomization	N/A

Reporting for specific materials, systems and methods

We require information from authors about some types of materials, experimental systems and methods used in many studies. Here, indicate whether each material, system or method listed is relevant to your study. If you are not sure if a list item applies to your research, read the appropriate section before selecting a response.

Materials & experimental systems

n/a	Involved in the study	
☒	☐	Antibodies
☒	☐	Eukaryotic cell lines
☒	☐	Palaeontology and archaeology
☒	☐	Animals and other organisms
☒	☐	Human research participants
☒	☐	Clinical data
☒	☐	Dual use research of concern

Methods

n/a	Involved in the study	
☒	☐	ChIP-seq
☒	☐	Flow cytometry
☒	☐	MRI-based neuroimaging
